# Focus on the Funding and Production of Evidence Rather Than Its Publication

**DOI:** 10.1371/journal.pmed.0020222

**Published:** 2005-07-26

**Authors:** Trevor Sheldon

**Affiliations:** **1**University of YorkYorkUnited Kingdom

Richard Smith has correctly highlighted the potential distortion of the evidence base caused by the publication of commercially sponsored trials [1]. However, his proposed solution could do with more thought.

First, let us be clear that the problem is possibly much wider than drug-company trials. There is the risk of systematic bias in reports of any research funded by a body that has an interest in the results. This “sponsor-induced bias” has been well documented in the area of tobacco-company-funded research on the effects of direct and indirect smoking [2]. In addition, governments, charities with an interest in a disease, and other bodies may also help to ensure that the results of research they sponsor (including trials) or the reporting of research favour one particular outcome. Lastly, individuals who carry out research, even if not funded by an interest group, may also bring prejudices to the table that influence the results and the published report. In other words, the tendency to bias is omnipresent. The issue of commercially funded trials is simply one of the degree and influence that trials have on clinical practice and health-care spending.

Following Smith's thinking to its logical conclusion, we would not publish any research but simply critiques. Smith proposes that instead of publishing trials, journals should concentrate on critically describing them. If he is not confident that the current system of peer review is sufficiently robust to identify weaknesses, why should he be any more confident in the critiquing process (which is a form of peer review)? Journal peer review is often ad hoc (especially when my work is rejected) and is in desperate need of professionalizing, but I suspect along with Smith that this is not sufficient protection. Surely the way to deal with the systematic risk of bias is a reform not in the publication but in the production of evidence, which in turn reflects the way it is funded, conducted, analysed, and reported.

My alternative solution in the case of trials is as follows. Companies (or indeed any body with a particular interest) should not be allowed to directly fund a clinical trial and no journal should publish a company-sponsored trial. Instead industry should pay a public or independent trials body, staffed by the best methodologists around and possibly established on an international scale. This international infrastructure should be publicly funded so that its staff do not feel dependent on industry business for security. The body, in conjunction with clinical experts from around the world, should conduct the study, ensuring that the questions are in the public's interest and fair (consumers would have an important role to play here).

This infrastructure would ensure that the research was of the highest standard and reported accurately. Once the funding had been agreed on, there would be a compulsion to register the trial and to publish no matter what the results. This body would also have the ability to carry out or commission economic modelling (which is even more susceptible than trials to sponsor-induced bias) [3]. The resulting data would be held in a publicly accessible data archive. We should have an international agreement that no phase III trials would be permitted other than through this route. While still a rather bureaucratic response, it would ensure that the evidence base was less contaminated. Drug companies might even find that such a social solution results in trials being cheaper and easier to run.
